# Splash M-knife versus Flush Knife BT in the technical outcomes of endoscopic submucosal dissection for early gastric cancer: a propensity score matching analysis

**DOI:** 10.1186/s12876-018-0763-5

**Published:** 2018-02-27

**Authors:** Mitsuru Esaki, Sho Suzuki, Yasuyo Hayashi, Azusa Yokoyama, Shuichi Abe, Taizo Hosokawa, Haruei Ogino, Hirotada Akiho, Eikichi Ihara, Yoshihiro Ogawa

**Affiliations:** 1 Division of Gastroenterology and Hepatology, Department of Medicine, Nihon University School of Medicine, 1-6 Kanda-Surugadai, Chiyoda-ku, Tokyo, 101-8309 Japan; 2 Department of Gastroenterology, Kitakyushu Municipal Medical Center, 2-1-1 Bashaku, Kokurakita-ku, Kitakyushu, Fukuoka, 802-0077 Japan; 3 Department of Medicine and Bioregulatory Science, Graduate School of Medical Sciences, Kyushu University, 3-1-1 Maidashi, Higashi-ku, Fukuoka, 812-8582 Japan

**Keywords:** Endoscopic submucosal dissection, Early gastric cancer, Hemostasis, Device, Splash M-knife

## Abstract

**Background:**

Endoscopic submucosal dissection (ESD) is a standard treatment for early gastric cancer. A new multi-functional ESD device was developed to achieve complete ESD with a single device. A metal plate attached to its distal sheath achieves better hemostasis during the procedure than the other needle-knife device, Flush Knife BT®, that has been conventionally used. The aim of this study was to compare the technical outcomes of ESD for early gastric cancer using the Splash M-Knife® with those using the Flush Knife BT.

**Methods:**

We conducted a retrospective review of the case records of 149 patients with early gastric cancer treated with ESD using the needle-type ESD knives between January 2012 and August 2016 at Kitakyushu Municipal Medical Center. Lesions treated with ESD using the Splash M-knife (ESD-M) and the Flush Knife BT (ESD-F) were compared. Multivariate analyses and propensity score matching were used to compensate for the differences in age, gender, underlying disease, antithrombotic drug use, lesion location, lesion position, macroscopic type, tumor size, presence of ulceration, operator level and types of electrosurgical unit used. The primary endpoint was the requirement to use hemostatic forceps in the two groups. The secondary endpoints of procedure time, en bloc and complete resection rates, and adverse events rates were evaluated for the two groups.

**Results:**

There were 73 patients in the ESD-M group, and 76 patients in the ESD-F group. Propensity score matching analysis created 45 matched pairs. Adjusted comparisons between the two groups showed a significantly lower usage rate of hemostatic forceps in the ESD-M group than in the ESD-F group (6.7% vs 84.4%, *p* < 0.001). Treatment outcomes showed an en bloc resection rate of 100% in both groups; complete resection rate of 95.6% vs 100%, *p* = 0.49; median procedure time of 74.0 min vs 71.0 min, *p* = 0.90; post-procedure bleeding of 2.2% vs 2.2%, *p* = 1, in the ESD-M and ESD-F groups, respectively. There were no perforations in either group.

**Conclusions:**

ESD-M appeared to reduce the usage of hemostatic forceps during ESD for early gastric cancer without increasing the adverse effects. Thus, it may contribute to a reduction in the total ESD cost.

## Background

Endoscopic submucosal dissection (ESD) has been widely accepted as a curative and minimally invasive treatment for gastric neoplasms [1–3]. However, bleeding during ESD remains a challenging complication, sometimes requiring replacement of devices to achieve hemostasis, thus increasing the difficulty of the procedure [4, 5]. In the event of bleeding during the ESD procedure, the endo-knife itself is first used to obtain hemostasis. However, if there is difficulty in achieving hemostasis, we switch to using hemostatic forceps such as Coagrasper® (Coagrasper, FD-410LR; Olympus, Tokyo, Japan), which costs 15,000 JPY (136 USD). In addition, frequent use of hemostatic forceps may be associated with hypercoagulation, which causes thermal damage to the gastric wall, leading to delayed perforation [6, 7]. In 2017, Tanaka et al. reported a 86% (57/66) usage rate of hemostatic forceps during ESD [8], reinforcing the difficulty in achieving hemostasis with the endo-knife alone.

**Fig. 1:**
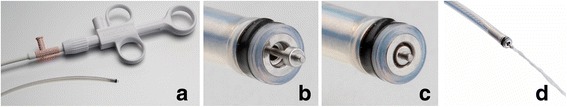
**a** Splash M-knife is a needle-type knife, with a needle length of 2.0 mm. **b** Open view of the tip of Splash M-knife. **c** Close view of the tip of Splash M-knife. A metal plate attached to the distal sheath helps hemostasis. **d** Splash M-knife has a water jet-assisted system to inject fluids through the tip of the knife

Recently, a new ESD device has been developed. Splash M-knife® (DN-D2718A; HOYA Corp., Pentax, Tokyo, Japan) (M-knife), or simply M-knife, is a new multi-functional ESD device, designed to achieve complete ESD with a single device. Pictures of M-knife are shown in Fig. 1a-d. It is a needle-type knife, with the most notable feature being its hemostatic ability. On closure of the M-knife, a metal plate attached to the distal sheath assists with hemostasis. If the entire procedure can be conducted using the M-knife alone without the usage of hemostatic forceps, it may result in consequent cost reduction. However, there are no reports on the hemostatic ability of the M-knife in ESD. Here we report the results of our evaluation of its efficacy in comparison with that of the conventional needle-type knife in achieving hemostasis during ESD.

## Methods

### Study design

This study was a single-center retrospective cohort study without randomization. We collected and retrospectively reviewed data from medical records, endoscopic reports, and the database of ESD at Kitakyushu Municipal Medical Center, Fukuoka, Japan.

### Patients

**Fig. 2:**
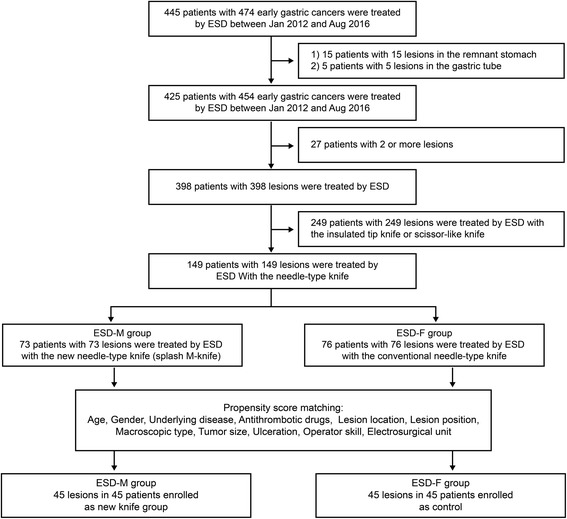
Flowchart of patients and lesions enrolled in this study. ESD, endoscopic submucosal dissection; ESD-M, endoscopic submucosal dissection with splash-M knife; ESD-F, endoscopic submucosal dissection with Flush Knife BT

Between January 2012 and August 2016, ESD for early gastric cancer was performed on 445 patients at the Kitakyushu Municipal Medical Center. We excluded 15 patients with lesions in the remnant stomach or the gastric tube. We also excluded 27 patients in whom two lesions were simultaneously treated by ESD. We further excluded 249 patients who were treated by ESD using the insulated-tip or scissor-type knife. Finally, 149 patients who underwent ESD either by Flush Knife BT or Splash M-knife were enrolled in the study. Flush Knife BT was mainly used from January 2012 to June 2015 while Splash M-knife was used from July 2015 to August 2016. The flowchart of patient selection is shown in Fig. 2.

### Method of ESD

#### ESD procedure

ESD-M was defined as ESD with M-knife, while ESD-F was defined as ESD with the conventional needle-type knife. We used Flush Knife BT (Fujifilm Co., Tokyo, Japan) 2.5 mm [9, 10] as the conventional needle-type knife. ESD for early gastric cancer (EGC) was performed using a standard technique, as described in detail elsewhere [1–3, 5]. Briefly, markings were placed around the lesion. Then, a mixture of equal parts of normal saline and 4% sodium hyaluronate with a small amount of epinephrine (0.001 mg/ml) and 0.8% indigo carmine was injected into the submucosal layer to lift up the lesion. After the injection, mucosal incision around the lesion and submucosal dissection were conducted with the Endo-knife. A VIO 300D or ICC 200 (ERBE Elektromedizin, GmbH, Tübingen, Germany) were used as the power sources for electrical cutting and coagulation. Marking and submucosal dissection were conducted in forced coagulation mode (30-40 W, effect; 2–3 in VIO300D, 40 W in ICC200). Mucosal incision was conducted in Endo Cut mode (effect 3, duration 3 in VIO300D, effect3 80 W in ICC200). We defined expert operators in this study as those who had experience in performing at least 50 cases of ESD for EGC, and trainees as those who had experience in performing less than 50 cases of that. Six experts and 13 trainees performed ESD in this study. All operators had used both knives at least 3 times before the study period, and therefore, were familiar with both the knives.

#### Hemostasis procedure

When bleeding or exposed vessels were detected, mainly during mucosal incision or submucosal dissection of the ESD procedure, hemostasis was carried out via coagulation with the tip of a needle-type knife. When we encountered any bleeding or detected any large vessels during ESD procedure, the initial hemostasis procedure was performed with the Endo-knife itself. We switched to use a hemostatic forceps when the initial three-minute hemostasis was not achieved. Coagrasper® (Soft coagulation mode; 80-100 W, effect; 5–6 in VIO300D, 80 W in ICC200) was the most frequently used hemostatic forceps in this study.

### Outcome

The primary endpoint in this study was the usage rate of the hemostatic forceps. The usage rate of the hemostatic forceps was defined as the percentage of lesions that required the use of hemostatic forceps at least once during the ESD procedure. According to the previous report, less than 20% of ESD using Flush Knife BT could be completed without the use of hemostatic forceps [8]. However, there were no data on the efficacy of M-knife in achieving hemostasis. We therefore hypothesized that 50% of ESD using M-knife could be completed without the use of hemostatic forceps, resulting in a 30% reduction in the usage of hemostatic forceps in this study. By assuming a 5% significance level and a statistical power of 80%, power analysis estimated that a minimum number of 39 patients would be required in each group. The secondary endpoints were procedure time for ESD, en bloc and complete resection rates, and adverse events rates (perforation and post-procedural bleeding). Procedure time for ESD was measured from the first injection after the mucosal marking to the completion of submucosal dissection. En bloc resection was defined as the whole tumor being removed in one-piece fashion. Complete resection was defined as the whole tumor being removed in one-piece fashion with tumor-free lateral and vertical margins. Perforation was diagnosed if extramural organ or fat protruding through the gastric wall was visualized during endoscopy, or if free air was seen on abdominal radiography or CT imaging. Post-procedural bleeding was diagnosed on the basis of clinical symptoms such as melena or hematemesis, or a decrease of > 2 g/dL in hemoglobin level after ESD, or bleeding proven with routine or emergency second-look endoscopy.

### Statistics

There were potential confounding biases between the two groups due to the retrospective nature of the study. In previous reports, including meta-analyses, large lesions, ulcerative (scar) lesions, lesions in the lesser curvature, lesions in the upper or middle position, flat or depressed morphology, the presence of gastric malignancy, male gender, antithrombotic drugs use, patients with underlying diseases (cardiomyopathy/cirrhosis/chronic kidney disease), and skill of operators were identified as prognostic factors for perioperative bleeding or technical difficulty of ESD [11–24]. Differences in these factors between the two groups might affect clinical outcomes. To compensate for these differences, we calculated propensity scores [25] using a logistic regression model (Fig. 2) for the following covariates: age (≧75 years vs < 75 years), gender (male vs female), underlying disease (none vs with cardiomyopathy or cirrhosis), antithrombotic drugs use (not receiving or discontinuation vs continuation), tumor size (≧21 mm vs < 21 mm), lesion location (in the upper or middle third of the stomach vs in the lower stomach), lesion position (in the lessor curvature of the stomach vs other positions), macroscopic type (flat or depressed vs protruding), ulceration (presence vs absence), skill of operators (expert vs trainee), and electrosurgical unit (VIO300D vs ICC200). Patients with similar propensity scores were matched in a 1:1 ratio from the ESD-M and ESD–F groups. Calipers (0.21) with a width equal to 0.25 of the standard deviation of the logit of the propensity score were used. We estimated the area under the receiver operating characteristics curve to validate the model in this study. The area under the curve was 0.744 and had predictive power. The two groups were evaluated in terms of the absolute standardized differences before and after matching to ensure balance of propensity scoring. Characteristics were considered to be in balance if the absolute standardized difference (ASD) was within 1.96√2/n after matching [26]. In this study, all ASDs were within 1.96√2/n after matching. To analyze the baseline characteristics of the patients and outcomes of this study, The Mann-Whitney U test or t test was used for continuous data, whereas the χ^2^ test and Fisher’s exact test were used for categorical data. Values of *p* < 0.05 were considered statistically significant for all tests. All analyses were performed using the JMP Pro 11.0 software.

### Ethics

This study was conducted in accordance with the Declaration of Helsinki. This study protocol was approved by the institutional review board of Kitakyushu Municipal Medical Center on November 18, 2016. Written informed consent was obtained from all patients before ESD.

## Results

### Baseline characteristics and outcomes before propensity score matching

The baseline characteristics of the 149 patients and lesions are shown in Table 1. ESD-M was conducted in 73 patients and ESD-F in 76. The percentage of expert operators was significantly higher in the ESD-M group than in the ESD-F group. There were no significant differences between the two groups with respect to other factors. Treatment outcomes before propensity matching are shown in Table 2.

**Table 1:** Baseline characteristics of the 149 patients who underwent ESD

	Total *n* = 149	ESD-M *n* = 73	ESD-F *n* = 76	*p* value	ASD
Age (years)					
Mean ± SD	73.3 ± 8.6	73.0 ± 8.4	73.6 ± 8.7	0.67^¶^	0.0702
Median (range)	74 (52–91)	74 (52–91)	74 (52–90)	0.67^§^	
Gender				0.4^‡^	
Male	97 (65.1%)	45 (61.6%)	52 (68.4%)		0.143
Female	52 (34.9%)	28 (38.4%)	24 (31.6%)		0.143
Underlying disease				0.49^‡^	
None	132 (88.6%)	66 (90.4%)	66 (86.8%)		0.113
Cardiomyopathy or cirrhosis	17 (11.4%)	7 (9.6%)	10 (13.2%)		0.143
Antithrombotic drugs				0.091^‡^	
None or discontinuation	143 (96.0%)	72 (98.6%)	71 (93.4%)		0.268
Continuation	6 (4.0%)	1 (1.4%)	5 (6.6%)		0.268
Location of lesions				0.055^†^	
Upper	24 (16.1%)	12 (16.4%)	12 (15.8%)		0.0163
Middle	54 (36.2%)	33 (45.2%)	21 (27.6%)		0.372
Lower	71 (47.7%)	28 (38.4%)	43 (56.6%)		0.371
Position of lesions				0.44^†^	
Lesser curvature	60 (40.2%)	30 (41.1%)	30 (39.5%)		0.0326
Greater curvature	38 (25.5%)	22 (30.1%)	16 (21.1%)		0.207
Anterior wall	29 (19.5%)	11 (15.1%)	18 (23.7%)		0.219
Posterior wall	22 (14.8%)	10 (13.7%)	12 (15.8%)		0.0592
Morphology				0.27^†^	
Protruding	55 (36.9%)	30 (41.1%)	25 (32.9%)		0.170
Flat	1 (0.7%)	1 (1.4%)	0 (0%)		0.169
Depressed	93 (63.4%)	42 (57.5%)	51 (67.1%)		0.199
Histology				0.68^‡^	
Differentiated	144 (96.6%)	70 (95.9%)	74 (97.4%)		0.0834
Undifferentiated	5 (3.4%)	3 (4.1%)	2 (2.6%)		0.0834
Lesion size (mm)					
Mean ± SD	16.6 ± 10.0	17.2 ± 9.3	16.1 ± 10.6	0.49^¶^	0.110
Median (range)	15 (3–77)	15 (3–40)	14 (4–77)	0.27^§^	
Specimen size (mm)					
Mean ± SD	39.0 ± 12.0	39.8 ± 11.6	38.2 ± 12.4	0.42^¶^	0.133
Median (range)	36 (17–110)	40 (17–75)	35 (20–110)	0.35^§^	
Depth				0.68^†^	
pT1a	132 (88.6%)	63 (86.3%)	69 (90.8%)		0.0724
pT1b1	7 (4.7%)	4 (5.5%)	3 (3.9%)		0.0757
pT1b2	10 (6.7%)	6 (8.2%)	4 (5.3%)		0.116
Presence of ulceration	17 (11.4%)	10 (13.7%)	7 (9.2%)	0.45^‡^	0.142
Operator level				< 0.001^‡^	
Experts	70 (47.0%)	47 (64.4%)	23 (30.3%)		0.727
Electrosurgical unit				0.009^‡^	
VIO 300D	128 (85.9%)	57 (78.1%)	71 (93.4%)		0.450
ICC200	21 (14.1%)	16 (21.9%)	5 (6.6%)		0.450

*ESD* endoscopic submucosal dissection, *ESD-M* endoscopic submucosal dissection with Splash-M knife, *ESD-F* endoscopic submucosal dissection with Flush Knife BT, *SD* standard deviation *ASD* absolute standardized difference

p value was calculated using the χ^2^ test ^†^ and Fisher’s exact test ^‡^ for categorical data

*p* value was calculated using the t test ^¶^ and the Mann-Whitney U test ^§^ for continuous data

pT1a, tumor invasion is within mucosa; pT1b1, tumor invasion is within 0.5 mm of the muscularis mucosae; pT1b2, tumor invasion is 0.5 mm or deeper into the muscularis mucosae

**Table 2:** Treatment outcomes of the ESD before propensity score matching

	Total *n* = 149	ESD-M *n* = 73	ESD-F *n* = 76	*p* value
Usage rate of hemostatic forceps	75 (50.3%)	10 (13.7%)	65 (85.5%)	< 0.001^* ‡^
Expert	28/70 (40.0%)	9/47 (19.1%)	19/23 (82.6%)	< 0.001^* ‡^
Trainee	47/79 (59.5%)	1/26 (3.8%)	46/53 (86.8%)	< 0.001^* ‡^
Procedure time(min)				
Mean ± SD	80.1 ± 51.1	83.8 ± 49.7	76.7 ± 52.4	0.4^¶^
Median(range)	70 (7–410)	74.0 (7–252)	69.5 (18–410)	0.3^§^
En bloc resection	149 (100%)	73 (100%)	76 (100%)	1^‡^
Complete resection	145 (97.3%)	70 (95.9%)	75 (98.7%)	0.36^‡^
Perforation	1 (0.7%)	0 (0%)	1 (1.3%)	1^‡^
Post-procedure bleeding	9 (6.0%)	4 (5.5%)	5 (6.6%)	1^‡^

*ESD* endoscopic submucosal dissection, *ESD-M* endoscopic submucosal dissection with Splash M-knife, *ESD-F* endoscopic submucosal dissection with Flush Knife BT, *SD* standard deviation

*p* value was calculated using Fisher’s exact test ^‡^for categorical data

*p* value was calculated using the t test ^¶^ and the Mann-Whitney U test ^§^ for continuous data

^*^significant value

En bloc resection was successfully performed in all patients from both groups. The overall rate of complete resection was 97.3%, with no significant difference between the two groups. Perforation occurred in only one patient in ESD-F. In contrast, post-procedural bleeding occurred in nine patients. Four of these were in ESD-M and the remaining five in ESD-F. Although three patients required blood transfusion, all bleeding was successfully controlled using endoscopic procedures with hemostatic forceps, and no patients required open surgical treatment. There were no treatment-related deaths. Of the 73 patients in the ESD-M group, hemostatic forceps were used during the ESD procedure in 10 patients (13.7%), compared with 65 of the 76 patients (85.5%) in the ESD-F group. Furthermore, hemostatic forceps were only required in one (3.8%) of the 26 ESD procedures performed by trainees.

### Treatment outcomes after propensity score matching

The matching factors between ESD-M and ESD-F after propensity score matching are shown in Table 3. Propensity score matching analysis created 45 matched pairs, which was an adequate number for statistical requirements. The treatment outcomes are shown in Table 4. Adjusted comparisons between the two groups showed a significantly lower usage rate of hemostatic forceps in the ESD-M group compared to the ESD-F group (6.7% vs 84.4%, *p* < 0.001). Treatment outcomes were similar in the two groups, (complete resection rate: 95.6% vs 100%, *p* = 0.49; median procedure time: 74.0 min vs 71.0 min, *p* = 0.90 in the ESD-M and ESD-F groups, respectively). With respect to adverse events, there were no perforations in either group; post-procedure bleeding was 2.2% vs 2.2%, *p* = 1 in the ESD-M and ESD-F groups, respectively; and there were no treatment-related deaths. The en bloc resection rate was 100% in both groups as before matching.

**Table 3:** Matching factors between the Splash M-knife and control groups after propensity score matching

	ESD-M *n* = 45	ESD-F *n* = 45	*p* value	ASD
Variable matching between groups				
Age, y; mean ± SD	73.5 ± 8.68	73.0 ± 8.95	0.802^¶^	0.0454
Gender; Male/Female	27/18	25/20	0.831^‡^	0.0464
Underlying disease; No/Yes	41/4	41/4	1^‡^	0
Antithrombotic drugs; No/Yes	44/1	44/1	1^‡^	0
Tumor size, mm mean ± SD	17.0 ± 9.46	16.8 ± 12.2	0.923^¶^	0.0183
Location of lesions; Upper or Middle/Lower	26/19	26/19	1^‡^	0
Position of lesions; Lesser curvature/others	19/26	18/27	1^‡^	0.0908
Macroscopic type; Flat or depressed/ protruding	27/18	29/16	0.828^‡^	0.0918
Presence of ulceration	4	4	1^‡^	0
Operator level; expert/trainee	22/23	22/23	1^‡^	0
Electrosurgical unit; VIO 300D/ICC 200	40/5	40/5	1^‡^	0

*ESD-M* endoscopic submucosal dissection with Splash M-knife, *ESD-F* endoscopic submucosal dissection with Flush Knife BT, *SD* standard deviation, *ASD* absolute standardized difference

*p* value was calculated using Fisher’s exact test ^‡^ for categorical data

*p* value was calculated using the t test ^¶^ for continuous data

**Table 4:** Treatment outcomes between the Splash M-knife and control groups after propensity score matching

	ESD-M *n* = 45	ESD-F *n* = 45	*p* value
Usage rate of hemostatic forceps	3 (6.7%)	38 (84.4%)	< 0.001^* ‡^
Procedure time(min)			
Mean ± SD	83.7 ± 48.5	84.2 ± 62.3	0.961^¶^
Median(range)	74.0 (21–240)	71.0 (18–410)	0.90^§^
En bloc resection	44 (100%)	44 (100%)	1^‡^
Complete resection	43 (95.6%)	44 (100%)	0.494^‡^
Perforation	0 (0%)	0 (0%)	1^‡^
Post-procedure bleeding	1 (2.2%)	1 (2.2%)	1^‡^

*ESD-M* endoscopic submucosal dissection with Splash M-knife, *ESD-F* endoscopic submucosal dissection with Flush Knife BT, *SD* standard deviation

*p* value was calculated using Fisher’s exact test ^‡^ for categorical data

*p* value was calculated using the t test ^¶^ and the Mann-Whitney U test ^§^ for continuous data

^*^significant value

## Discussion

To our knowledge, this is the first report describing the efficacy of this new needle-type device, the M-knife, compared to that of Flush Knife BT which is one of the conventional needle-type knives. ESD with M-knife was associated with a significantly lower usage rate of hemostatic forceps. In addition, ESD with M-knife achieved similar technical outcomes to those of Flush Knife BT with no increase in adverse events.

There are two conventional types of knives used in ESD: the needle-type knife and the insulated-tip knife [27]. In the needle-type knives, there were two major developments before the advent of the M-knife. One was the water jet-assisted system, which allowed injection of fluids through the tip of knife during ESD procedure. This allowed for fluid injection without changing instruments, significantly shortening the procedure time [28–30]. The other development was the attachment of a ball-tip to the tip of the knife, improving its operability and increasing hemostatic efficacy. In this study, we used the Flush Knife BT, which has both of these features, as a conventional needle-type knife [9, 10]. The ball-tip enables easy removal of tissues during incision or dissection. Furthermore, by attaching a ball-tip, the diameter of the knife is expanded up to 0.9 mm, facilitating hemostasis. Although Flush Knife BT improves hemostatic efficacy with these two developments compared to standard Flush Knife, we still sometimes encountered cases wherein hemostasis was unsuccessful using this endo-knife alone, and consequently, hemostatic forceps were required [8, 10].

The M-knife, which also has the above two features, is developed to overcome the problem of hemostasis. A metal plate, 1.8 mm in diameter, is attached to the distal sheath of this knife. This is twice as long as the ball-tip of the Flush Knife BT, and the contact area of the metal plate is four times larger. When the M-knife is closed during the procedure, this metal plate facilitates hemostasis. The significantly different outcome in terms of the hemostatic ability of these 2 knives in this study was thought to be due to the difference in the device structure.

In this study, we selected the usage rate of hemostatic forceps as a primary endpoint to evaluate the hemostatic ability of the ESD devices. The 86% usage rate of hemostatic forceps reported by Tanaka et al. [8] is similar to that in the Flush Knife BT group in this study. In contrast, ESD was completed with a single knife without hemostatic forceps in 86.7% of patients in the M-knife group. Although ESD has the benefit of a high en bloc resection rate even for larger or ulcerative lesions in any part of the stomach, as well as accurate pathological evaluation [1–3], the cost of ESD is higher than that of endoscopic mucosal resection (EMR). This is one of the disadvantages of ESD in the treatment of early gastric cancer compared to EMR. The cost of the Coagrasper® hemostatic forceps is about 15,000 yen. A 50% reduction in its use would result in a less costly hemostasis during ESD procedure. Using the M-knife may thus result in a reduction of total ESD cost due to a lower usage rate of hemostatic forceps.

We did not restrict the number of hemostatic attempts with the needle-type knife, although other studies have done so [4]. Thus, the number of hemostatic attempts with the M-knife may have been greater than that in the conventional needle-type knife group. However, neither procedure time nor perforation rate was significantly different between the two groups. Hemostatic attempts with the endo-knife alone seems to have few disadvantages either in the length of procedure time or risk of perforation.

We included patients treated not only by experts but also by trainees in this study. Before matching, trainees conducted 35% (26/73) of ESD with M-knife and hemostatic forceps was used in only one case. Therefore, no special skill is required for hemostasis with the M-knife, which is useful not only for experts but also for trainees.

This study has several limitations. Firstly, this was not a prospective randomized controlled trial. It thus has the limitations of retrospective studies, as all relevant data may not be available in detail, even though we conducted a propensity score matching analysis. Secondly, this study was conducted in a single center with a restricted number of study samples. A multicenter trial will be performed to determine the advantage of using the M-knife during ESD procedure in the near future. Thirdly, Flush Knife BT was used in the initial phase of this study while Splash M-knife was used during the late phase. The possibility that an institutional learning curve, if any, that could affect the outcomes of the study cannot be denied. Fourthly, the initial hemostasis procedure was performed with Endo-knife. We switched to use a hemostatic forceps when the initial three-minute hemostasis was not achieved. This might be affected by certain factors including the operator’s preference, experience and skill and the size of the vessels; some of which might not be fully compensated for by the propensity score matching analysis. Finally, this study compared the efficacy of the M-knife to that of the Flush Knife BT, which is one of the conventional needle-type knives. Further studies are required to compare clinical outcomes of M-knife not only to other needle-type knives but also to other type of endo-knives including the insulated-tip knife and grasping-type knife [31, 32].

## Conclusions

Compared to the Flush Knife BT, Splash-M knife appeared to reduce the usage of hemostatic forceps during ESD procedure with no increase in adverse events. Thus, it may contribute to a reduction in the total ESD cost. Furthermore, ESD with Splash M-knife is feasible even for trainees.

## Abbreviations

ASD: Absolute standardized difference
EGC: Early gastric cancer
ESD: Endoscopic submucosal dissection
ESD-F: Endoscopic submucosal dissection with Flush Knife BT
ESD-M: Endoscopic submucosal dissection with M-knife
M-knife: Splash M-knife®
SD: Standard deviation
